# Oil-Impregnated Hydrocarbon-Based Polymer Films

**DOI:** 10.1038/s41598-018-29823-7

**Published:** 2018-08-03

**Authors:** Ranit Mukherjee, Mohammad Habibi, Ziad T. Rashed, Otacilio Berbert, Xiangke Shi, Jonathan B. Boreyko

**Affiliations:** 1 Macromolecules Innovation Institute, Department of Biomedical Engineering and Mechanics, Virginia Tech, Blacksburg, Virginia 24061 USA; 2 Bemis North America, Neenah, Wisconsin 54957 USA

## Abstract

Porous surfaces impregnated with a liquid lubricant exhibit minimal contact angle hysteresis with immiscible test liquids, rendering them ideal as self-cleaning materials. Rather than roughening a solid substrate, an increasingly popular choice is to use an absorbent polymer as the “porous” material. However, to date the polymer choices have been limited to expensive silicone-based polymers or complex assemblies of polymer multilayers on functionalized surfaces. In this paper, we show that hydrocarbon-based polymer films such as polyethylene can be stably impregnated with chemically compatible vegetable oils, without requiring any surface treatment. These oil-impregnated hydrocarbon-based films exhibit minimal contact angle hysteresis for a wide variety of test products including water, ketchup, and yogurt. Our oil-impregnated films remain slippery even after several weeks of being submerged in ketchup, illustrating their extreme durability. We expect that the simple and cost-effective nature of our slippery hydrocarbon-based films will make them useful for industrial packaging applications.

## Introduction

Slippery liquid-infused porous surfaces (SLIPS) exhibit remarkable properties such as ultra-low contact angle hysteresis (CAH) for a wide variety of liquids^1,2^, excellent self-healing capability^2,3^, and stability under high pressures or temperatures^2,4,5^. In addition to repelling liquids, SLIPS have been shown to promote anti-fouling^6–13^, self-cleaning^1,2,14^, anti-icing^15–20^, reduced drag^21–24^ and enhanced phase-change heat transfer^25–31^.

Two primary criteria must be met to ensure stable SLIPS: (1) The surface must have nano/micro-roughness which can hold the lubricant in place by capillary action, and (2) The substrate must have a greater chemical affinity for the lubricant than the working fluid it is repelling^1,2^. The substrate can either be an impermeable material that is roughened and/or porous^7,32–41^ or an absorbent polymer where the “nano-roughness” is the molecular matrix itself^3,9,14,42–48^.

Absorbent polymer coatings are an increasingly popular choice for SLIPS because of their compatibility with a wide variety of industrial metals and practical materials^14^. However, so far the polymer choices have been limited to expensive silicone or fluorine-based polymers^3,9,39,42,43^ and/or involve the complex assembly of multi-layer polymer coatings on functionalized surfaces^46–48^. There is also a class of cross-linked elastomers called organogels or fluorogels which can be swollen with oil^14,44,49,50^, which are practically limited by similar issues of cost and complexity. Organogels have also been cured with oil inside, but this approach requires a change in environment (heat or pressure) to release the oil toward the surface^51^.

Hydrocarbon-based polymers such as polyethylene (PE) and polypropylene (PP) are the most common plastics in the world in terms of production volume and are widely used for packaging applications due to their cost effectiveness and chemical resistance^52^. Despite the obvious attraction of converting extruded film packaging directly into SLIPS to maximize product drainage, to date this has not been considered because hydrocarbon-based films like PE are generally considered resistant to impregnation by oils. Indeed, PE has even been used as a backing material for SLIPS specifically because of its superb chemical and moisture resistance^53^. While oil has been impregnated within microporous PP membranes, the oil was impregnating the micropores not the PP itself^39^.

Long before the invention of SLIPS, there has been a technique of mixing a molten solution of ultra-high molecular weight (UHMW) polyethylene with an oil, typically for use as roller bearings^54,55^. However, this is fundamentally different from SLIPS in two important ways. First, this approach requires the oil to be mixed with the molten polymer prior to curing, whereas for SLIPS the lubricant is simply wicked into an already-made solid substrate. Second, the oil cured within the polymer using the molten method cannot freely migrate to the outer surface unless subjected to a strong force, such as the centrifugal force experienced by bearings. This is in sharp contrast to the impregnated liquid of SLIPS, which can passively wick to the outer surface to replenish any lubricant that has been depleted over time. Similarly, Golovin *et al*. has reported plasticizing hydrocarbon-based polymers with oils, in order to tune the mechanical properties of the polymer to minimize ice adhesion^56,57^. In short, to date there has not been any demonstration of using hydrocarbon-based polymers as SLIPS.

Here, we present the surprising finding that several extruded hydrocarbon-based polymer films such as polyethylene can be directly and stably impregnated with lubricating oil to convert them into SLIPS. Impregnation was enabled by utilizing low density polymers, such as ultra-low density polyethylene (ULDPE), in conjunction with vegetable oils that exhibit excellent chemical compatibility with the polymer film. While the wicking rate of the oil into hydrocarbon-based polymers was indeed extremely slow, we demonstrate that this is not problematic in the context of impregnating micrometric film layers. Finally, we show that our hydrocarbon-based SLIPS can durably repel both Newtonian fluids (water) and non-Newtonian fluids (ketchup and yogurt), making them ideal for a variety of packaging applications.

## Results

### Oil-Impregnation of ULDPE Films

**Figure 1:**
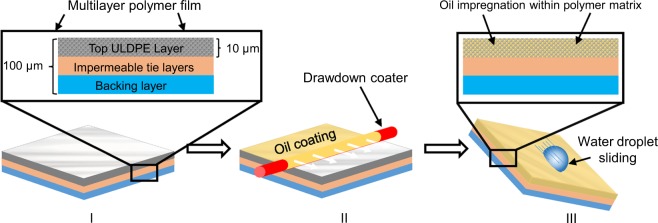
Schematic of how carbon-based polymer films such as ULDPE can be easily modified to become slippery oil-impregnated surfaces. (**I**) A multilayer extruded polymer film is used as substrate, with ULDPE as the top layer. (**II**) A small, controlled volume of oil is spread across the face of the ULDPE layer using a drawdown coater. (**III**) Chemically compatible oils easily impregnate within the thin ULDPE layer to create a durable, slippery surface.

To demonstrate how practical extruded films can be easily converted into SLIPS, we used a drawdown coater to impregnate oil into the top layer of a multilayer commercial-grade polymer film (Fig. 1). As shown in Fig. 1, the rod in the drawdown coater moved over a dry film at a constant velocity with a trace amount of oil and ensured that a smooth oil layer is formed over the film. For this initial proof-of-concept, the top layer of the film was approximately 10 *μ*m thick and comprised of ultra low-density polyethylene (ULDPE), while the tie layers were highly impermeable to the oil in order to isolate the SLIPS to within the top layer (see Experimental Section for more information). After rod-coating the top ULDPE layer of the film with a small amount of cottonseed oil, it was observed that the surface became extra slippery to deposited water droplets (i.e. low CAH) as quantified in the proceeding section.

Of course, it is possible that the slippery properties of the film were merely due to a bulk layer of oil resting atop the surface, as opposed to the oil actually impregnating within the ULDPE. This distinction is not trivial: oil in the former case is easily sheared off due to gravity or other forces, while oil for the latter case is locked within the interstitial spaces between the polymer molecules for excellent stability^21,58,59^. Using gravimetric measurements^34,35^, the total oil amount spread across the sample after rod-coating was found to be about 1.9 g/m^2^. Absorbent wipes were then used to firmly remove the excess oil resting atop the film. The 1.2 g/m^2^ of oil removed by the wipes corresponded to an initial excess oil layer atop the film whose thickness was approximately 1 *μ*m. Even after removing the excess, 0.7 g/m^2^ of oil remained impregnated somewhere in the ULDPE layer. The rod-coated films remained equivalently slippery even after the excess oil was removed, which further indicates that the oil is indeed impregnating the ULDPE. The similar contact angle hysteresis of droplets on impregnated films with/without an excess layer agrees with a recent study by Muschi *et al*., who showed that the excess layer does not tend to affect the slippery properties of SLIPS^60^.

**Figure 2:**
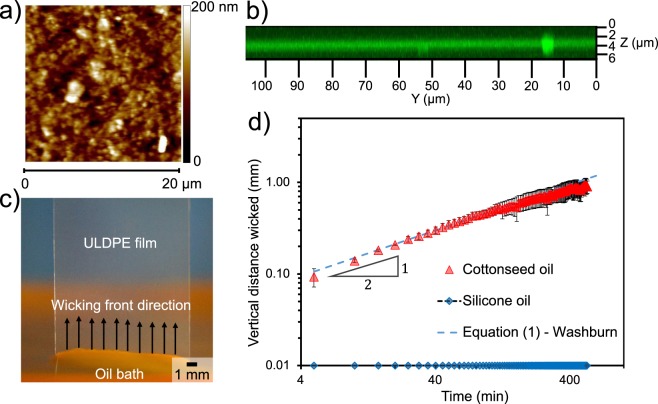
(**a**) Topographical map of the surface roughness of a dry ULDPE film, as obtained by atomic force microscopy over a scan area of 20 *μ*m × 20 *μ*m. (**b**) Image stack of an oil-impregnated ULDPE film, as obtained by laser scanning confocal microscopy. The bright green band represents fluorescently dyed cottonseed oil, which impregnated the ULDPE film to a depth of about 1.3 *μ*m. Excess oil atop the film was removed prior to imaging. (**c**) Photograph of the wicking setup, where a vertical ULDPE film is partially submerged in a fluorescent-dyed bath of cottonseed oil. For oil choices where wicking is possible, an advancing front progresses up the film by capillary action (black arrows) (Supplementary Movie M1). (**d**) Plot of the vertical displacement of the oil over a time span of 9 hr. The advancing front of the cottonseed oil followed Washburn’s law (red triangles), while no wicking was observed when using silicone oil (blue diamonds). For this graph and all future graphs, each data point represents an average from three trials, while the error bars correspond to plus or minus a standard deviation.

After removing the excess oil, where is the remaining oil residing? One possibility is that the exterior of the ULDPE layer exhibits a surface roughness, capable of impregnating the oil. Atomic force microscopy (AFM) revealed a root mean square roughness of only 21.4 nm (Fig. 2a). In contrast, laser scanning confocal microscopy revealed that the oil was able to impregnate to a depth of 1.3 *μ*m (Fig. 2b). These findings therefore confirm that the oil is impregnated within the bulk of the ULDPE layer, as opposed to merely residing within the exterior surface roughness.

To be absolutely certain that oil impregnation is occurring, the rate of oil wicking across the ULDPE was characterized. The film was oriented vertically and its bottom end was submerged into an oil reservoir (Fig. 2c). This setup ensures that the propagation of oil up the film is solely due to impregnation (i.e. wicking) and cannot be caused by gravitational spreading^61–63^. A small amount of fluorescent dye was added to the oil reservoir to assist the imaging of the advancing oil front up the ULDPE film.

Figure 2d graphs the displacement of the oil front over time. When using cottonseed oil as the working fluid, the oil displacement followed the well-known Washburn equation that balances capillary action against viscous dissipation^64^:

1 $$l(t)=\sqrt{\frac{{\gamma }_{o}rt}{2{\mu }_{o}}},$$

where *γ*_*o*_ = 0.03 N/m and *μ*_*o*_ = 0.07 kg/m-s are the surface tension and viscosity of the cottonseed oil, respectively, and *r* is the effective “pore” radius of the interstitial gaps between ULDPE molecules. The excellent fit of the data to the $l\sim {t}^{\mathrm{1/2}}$ power-law slope of Equation (1) confirms that the oil is able to impregnate inside of the molecular spaces of ULDPE. This finding is notable given that polyolefins like PE are well-known for their chemical/moisture resistance and never before used for SLIPS. Furthermore, the effective pore radius that obtains a best fit of Equation (1) to the data, *r* ≈ 0.175 nm, is a good match to reports measuring the interstitial spacing between PE molecules to be about 0.2 nm^65,66^. This small value of *r* results in extremely slow wicking rates, for example it takes roughly 10 hr for the cottonseed oil to impregnate a mere 1 mm into the ULDPE! However, we emphasize that this does not matter in the context of infusing ultra-thin extruded films. For example, if the oil is spread uniformly across the top face of the film, it will only take about *t* ≈ 0.05 s to completely impregnate the 1.3 *μ*m thickness within the ULDPE layer. While there has been some debate on the applicability of the Washburn equation at the nanoscale^67,68^, recently it has been shown that the Washburn equation holds even when the pore radius is only 10 times the size of a liquid molecule^69^. This previous finding, along with the excellent fit of our wicking dynamics to the 1/2 power law, validate our use of the classical Washburn equation.

The wicking of cottonseed oil within the ULDPE film confirms that the nanometric intermolecular spaces can hold the oil layer by capillary action, which satisfies the first criteria required for stable SLIPS. This first criteria is further validated by side-view imaging of a 10 *μ*L cottonseed oil droplet on ULDPE, which revealed a static contact angle of *θ*_*o*_ ≈ 43° < 90°. Recall that the second criteria dictates the impregnated oil layer should not be displaced by a deposited test liquid. As shown by Lafuma and Quéré, the oil should not get displaced from a thermodynamic standpoint when the following inequality is satisfied^1^:

2 $${\gamma }_{o}cos{\theta }_{o}-{\gamma }_{w}cos{\theta }_{w}-{\gamma }_{o/w} > \mathrm{0,}$$

where subscripts ‘o’ and ‘w’ denote the oil and water phases, respectively. Using the pendant droplet method on a goniometer, the value of the oil-water surface tension was measured to be *γ*_*o*/*w*_ = 0.021 N/m. After measuring *θ*_*w*_ ≈ 97° on ULDPE, the left-hand side of Equation (2) becomes approximately 10 mN/m which satisfies the stability criteria.

As both capillary wicking and swelling in polymers are diffusion-controlled processes which follow the familiar 1/2 power law, it is possible that the oil imbibition in the ULDPE film is actually a swelling mechanism which will eventually degrade the polymer. The Plastics Design Library (PDL) is a resource that exhaustively details the interactions of different polymer grades with a variety of mediums^70^. The ULDPE films used in this study are essentially linear low density polyethylenes (LLDPE). The PDL assigns a rating of 9 for the specific combination of LLDPE in a cottonseed oil medium which corresponds to a weight change of less than 0.5% for the LLDPE. So we can safely assume negligible swelling in the ULDPE films used here, as the mass of cottonseed oil used to infuse the ULDPE was typically only about 2 g/m^2^. In general, such limited swelling in polymers follows first-order diffusion kinetics (i.e. $\sim {t}^{1}$ dependence) in contrast to the second-order diffusion kinetics ($\sim {t}^{\mathrm{1/2}}$) during severe swelling^71^. But our wicking test revealed that the oil front and consequently the mass uptake amount is following second-order kinetics without any appreciable swelling of the ULDPE film. Thus, the primary mechanism of the oil impregnation within the polymer is the capillary imbibition of the oil inside the amorphous regions of the polymer, rather than swelling of the polymer.

In addition to cottonseed oil, we also found that canola oil and soybean oil successfully impregnated the ULDPE films as evidenced by the reduced CAH of deposited water droplets (Supplementary Figure S1). This suggests that vegetable oils in general are chemically compatible with the ULDPE films. To illustrate the importance of chemical compatibility, we repeated the wicking test with silicone oil which is a synthetic oil. Even after 9 hr of the ULDPE film being in contact with the oil reservoir, there was absolutely no wicking front observed (Fig. 2d). This chemical compatibility between different oils and ULDPE can be explained by means of their molecular structures. Permeation of liquids in polymers can be affected by the permeant’s molecular weight and polarity, as well as the polymer’s free volume. From the chemical composition of cottonseed oil^72^ we found out the average molecular weight of cottonseed oil is about 870 g/mol which is similar to the molecular weight of 10 cSt silicone oil^73^. Thus, the primary factor that affects permeation into ULDPE is most likely their polarity. A polar permeant will in general have a higher affinity for permeating in a polar polymer than in a non-polar polymer and vice versa^74^. Natural oils and fats are composed of complex mixtures of non-polar triglycerides, whereas silicone oil (polydimethylsiloxane) has a polar characteristic due to the presence of polar Si−O bonds^75^ (Supplementary Figure S2). This explains why non-polar vegetable oils wick inside non-polar ULDPE while polar silicone oils do not.

In general, we note that existing reports of SLIPS utilize polymers that are already well-known to be absorbent, while avoiding materials like ULDPE that are assumed to be impermeable. As revealed by our surprising test results with ULDPE, we suggest that wicking tests should be conducted with a wide variety of material and oil combinations. This could not only reveal an expanding palette of material choices that are suitable for SLIPS, but even reveal which oils are able to impregnate a given material.

### Wetting Properties of Oil-Impregnated Films

**Figure 3:**
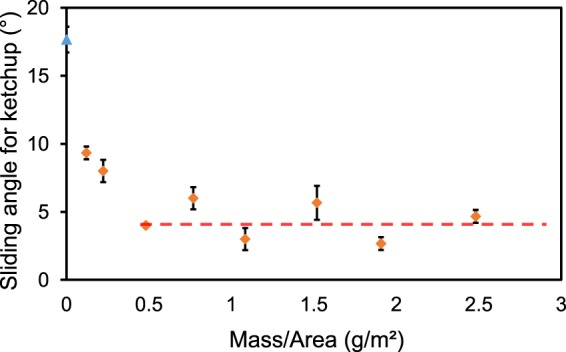
The sliding angle for a dollop of ketchup (1.3 g) as a function of the oil amount applied to a ULDPE film. The dashed red line indicates that the tilt angle becomes constant at about 4° beyond an oil uptake of 0.5 g/m^2^. For comparison, the sliding angle on a dry ULDPE film is indicated by the blue triangle.

What is the minimum amount of impregnated oil required to preserve the maximal slipperiness of the ULDPE? To find the lower limit, a set of experiments was performed with various mixtures of oil and isopropyl alcohol. The isopropyl alcohol rapidly evaporated after coating the ULDPE film, resulting in oil uptake amounts of any desired value based on the mixture ratio. We used ketchup “droplets” instead of water droplets to test the slipperiness of surfaces, as ketchup exhibits larger values of hysteresis making it easier to detect variations in surface friction with changing oil amounts. Ketchup is too viscous for the shrink-swell method of CAH measurement; instead, a motorized tilt base was used to find a critical sliding angle (SA) for a fixed mass of ketchup (1.3 g). Figure 3 shows the results, where now the SA (and by extension the hysteresis) can clearly be seen to significantly decrease with increasing oil uptake until reaching a minimal value at a critical concentration of about 0.5 g/m^2^. Any further increase in oil uptake amount does not significantly affect the SA. This critical uptake amount is in good agreement with the aforementioned gravimetric measurements, which revealed that about 0.7 g/m^2^ of oil is able to actually impregnate the ULDPE. These results clearly demonstrate that only an extremely small amount of oil is required to maximize the slipperiness of the impregnated ULDPE films. For the rest of the paper, an oil uptake amount of about 2.3 g/m^2^ was used, as this is above the lower limit while also removing the need to add any isopropyl alcohol for our particular drawdown coater.

**Figure 4:**
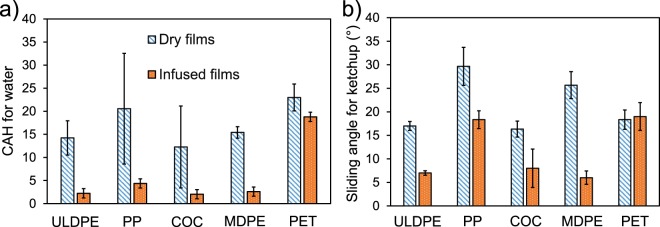
(**a**) The contact angle hysteresis of water droplets on five different polymer films: ultra-low density polyethylene (ULDPE), polypropylene (PP), cyclic olefin copolymer (COC), medium-density polyethylene (MDPE), and polyethylene terephthalate (PET). (**b**) The sliding angle of a 1.5 g dollop of ketchup on the same films. In both charts, blue bars represent the CAH when the polymers were dry and untreated, while the orange bars represent the same polymers but impregnated with cottonseed oil using a drawdown coater.

Another germane question: besides ULDPE, can any other hydrocarbon-based polymer films be impregnated with oil? To answer this question, the slipperiness of various types of commercial-grade extruded films were characterized, comparing dry films to equivalent films impregnated with cottonseed oil. For dry polymer films, the hysteresis of water droplets is always above CAH > 10° (Fig. 4a). Upon oil impregnation, four of the five films: ULDPE, polypropylene (PP), cyclic olefin copolymer (COC), and medium-density polyethylene (MDPE) exhibited a significant decrease in hysteresis to CAH < 5°. This reveals that beyond just ULDPE, many types of hydrocarbon-based polymer films are suitable for creating SLIPS. Similar results were obtained with 1.5 g dollops of ketchup, where the sliding angle was dramatically reduced for the same four polymer films (Fig. 4). However, only the oil-impregnated ULDPE, COC, and MDPE were able to produce sliding angles of SA < 10°. The sliding angle of the infused PP was closer to SA ≈ 18°, although this was still only half the value of the equivalently dry PP (SA ≈ 30°). We will now return to using ULDPE films for the remainder of this report, but clearly MDPE and COC (and to a lesser extent, PP) are also suitable candidates for SLIPS.

Why do polymers like ULDPE and COC facilitate oil impregnation while PET does not? We suggest this is a result of differing free volumes in the polymers. Free volume corresponds to the amount of amorphous phase or degree of crystallinity in a polymer. Permeation of liquids or gases takes place only in the amorphous phase within the polymer^76^ and in general increases with increasing free volume, as shown by Lee^77^. The degree of crystallinity is only 16% for ULDPE and 2% for COC, indicating these films are predominantly amorphous. Apart from the degree of crystallinity, the polarity of the polymer and permeant molecules also affects the penetration of a liquid into a polymer. Due to the presence of oxygen containing functional group, PET has a high level of polarity. Thus, it is much harder for non-polar vegetable oils (Supplementary Figure S2) to permeate into PET than in non-polar ULDPE or COC polymer^74,78^. It is also possible that PET has a higher degree of crystallinity, but this information was proprietary for this particular resin product.

**Figure 5:**
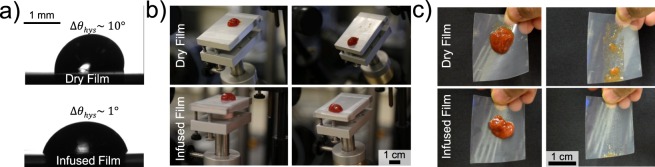
(**a**) Water droplets sliding down dry ULDPE films have a noticeable asymmetry in shape due to CAH (top), while there is no appreciable difference between the advancing and receding angles of droplets on oil-impregnated ULDPE (bottom). (**b**) The sliding angle of a 1.3 g dollop of ketchup is 23° on dry ULDPE (top images), but only 4° on oil-impregnated ULDPE (bottom). (**c**) On dry films (top images), sliding ketchup leaves behind considerably more residue than on infused films (bottom).

Using the tilt method, snapshots of sliding water droplets were taken to visually capture the difference in CAH of dry versus oil-impregnated ULDPE (Fig. 5a). On dry ULDPE, droplets exhibit CAH∼10°, such that the droplet shape is obviously tilted toward its advancing contact line. In contrast, the oil-impregnated ULDPE exhibits CAH∼1°, such that sliding droplets do not have any appreciable shape change. Figure 5b illustrates the dramatic reduction in sliding angle for ketchup on the oil-impregnated ULDPE, where a small dollop of ketchup can easily slide at low angles (SA ≈ 5°) without having to dramatically change its shape. Finally, by holding vertically oriented films against the light, it can be seen that ketchup sliding down an oil-impregnated ULDPE film leaves much less residue on the film compared to dry ULDPE (Fig. 5c).

### Durability Tests

When long sheets of extruded polymer films are manufactured, they are commonly rolled up for storage prior to distribution. In this rolled-up configuration, the top layer of the multi-layer film is firmly pressed against the bottom layer. In packaging applications, only the top (i.e. inside) layer should be oil-impregnated, so it is important to know whether there will be an undesirable transfer of oil from the top layer to the bottom layer during storage. This was tested by stacking four multi-layer extruded films on top of each other, where both the top layer and bottom layer of each film were ULDPE but only the top layers were oil-impregnated. A weight of 5 kg was placed on top of the stack, such that the three bottom films all had an oil-infused ULDPE layer that was firmly pressed against the dry ULDPE layer of the next film.

**Figure 6:**
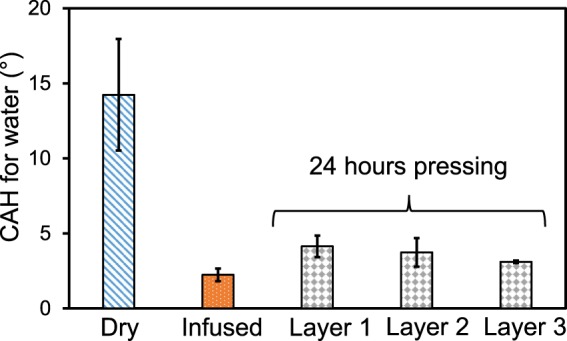
The physical stability of the oil-impregnated ULDPE was demonstrated by pressing 3 layers of oil-infused ULDPE against dry layers of ULDPE with a 5 kg weight. Even after 24 hr of pressing, the CAH of the oil-impregnated ULDPE layers remained extremely low (<5°), indicative of stable SLIPS.

After 24 hr of pressing the films together, the weight was removed and the CAH of water droplets was measured on the top face of each of the three films. Figure 6 shows that after 24 hr of pressing, the hysteresis of the water increased only by 1–2°, remaining under CAH < 5° as desired for SLIPS. Most notably, this is still an order of magnitude reduction in CAH compared to the dye ULDPE films. This indicates that only a very small amount of oil drained from the top layer during pressing against the bottom layer, with the large majority of the oil remained stably locked within the top layer.

### Drainage from Oil-Impregnated Pouches

**Figure 7:**
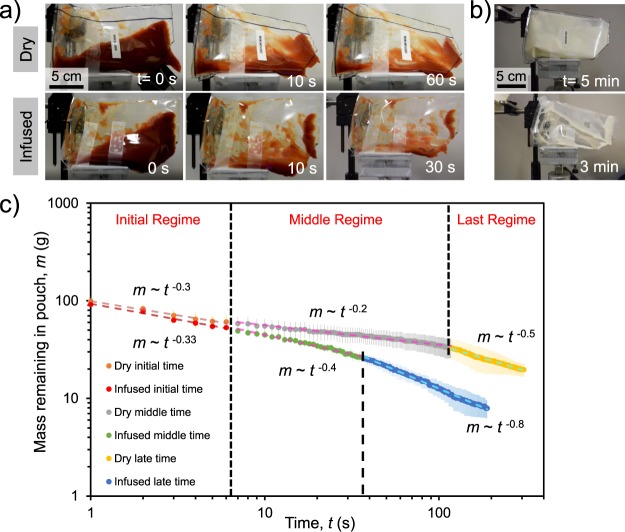
(**a**) Time-lapse of ketchup draining from dry (top) and oil-impregnated (bottom) ULDPE pouches using a tilt angle of *α* = 45° (camera is also tilted). After 60 s of drainage, a large amount of ketchup is still sticking to the walls of the dry pouch, while the infused pouch is almost completely cleaned out after only 30 s (see Supplementary Movie M2). (**b**) For yogurt drainage, even after 5 min of drainage there is much more yogurt trapped in a dry ULDPE pouch (top) compared to an oil-impregnated pouch (bottom) after only 3 min. (**c**) Logarithmic plot of the ketchup mass remaining in the pouch versus time. Vertical dashed lines demarcate three different power-law regimes. For the second and third regimes, the power-law slope was more pronounced for the oil-impregnated pouch.

To test the effects of oil-impregnation on product drainage, we created three-dimensional open pouches (dimensions: 18 cm × 12 cm × 6 cm) by bonding together five films with an impulse sealer (see Experimental Section 4.4). Equivalent pouches were made from dry or oil-impregnated ULDPE films, filled with 170 g of ketchup, and then poured at a 45° angle using the goniometer’s motorized tilt base. For the first 10 s of pouring, the drainage rates of the ketchup were similar for both the dry and oil-impregnated ULDPE. Beyond 10 s, however, the oil-impregnated pouch drained ketchup at a significantly faster rate than the dry pouch and also minimized how much residue was stuck to the films at the end of pouring (Fig. 7a and Supplementary Movie M2). Similar results were obtained with yogurt (Fig. 7b).

The enhanced drainage of ketchup from oil-impregnated pouches was preserved even when the ketchup-filled pouches were stored for over 50 days prior to drainage (Supplementary Figure S3). This long-term durability of our infused pouches indicates that there is negligible chemical interaction between the lubricating oil layer and ketchup over time. Moreover, it has been found that for a constant average flow velocity, the depletion of the lubricating layer is delayed if the the viscosity of the external fluid is much greater than the viscosity of the lubricating oil due to reduced interfacial velocity^79^. Given ${\mu }_{{\rm{ketchup}}}\gg {\mu }_{{\rm{oil}}}$ and the short drainage time, we can safely attest that the shear force exerted by the bulk ketchup or any other viscous food product will have negligible effects on the durability of the infusion.

**Figure 8:**
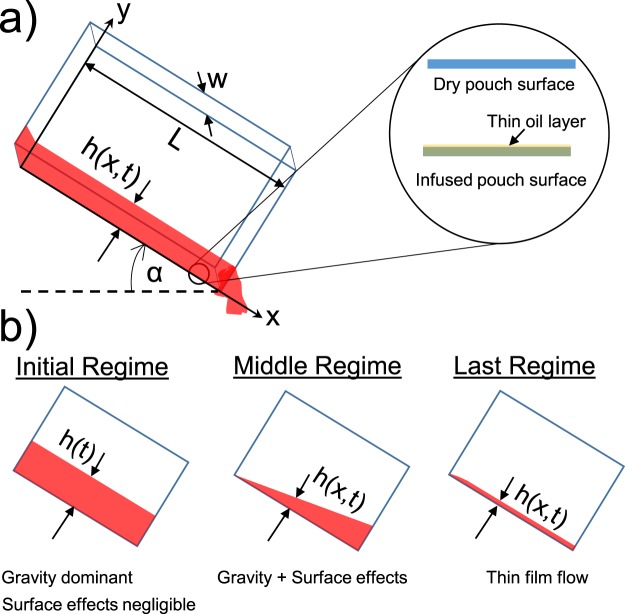
(**a**) Schematic of the pouch drainage: *w* and *L* denote the width and length of the rectangular pouch respectively, *h* is the height of ketchup which can be function of position and time, and *α* is the inclination angle. The inset depicts the difference between dry and oil-impregnated polymer films comprising the pouch. (**b**) Schematic of the three drainage regimes, where the primary difference is the height profile.

To quantify the drainage rates, the ketchup was poured into a container placed on a digital mass balance to measure how much ketchup was still in the pouch at any given time. The oil-impregnated surface was able to drain almost 90% of the ketchup in about 50 s, which is only 1/6 of the time required for the dry pouch to drain the same amount. By plotting the drainage over time in a logarithmic plot (Fig. 7c), we identified three distinct power-law regimes of drainage as illustrated in Fig. 8. These power laws can be independently rationalized using scaling analysis, provided that the following assumptions are made: (i) Pouch deformation is neglected, such that the ketchup flows out of a rigid rectangular opening of constant width *w*. The mass remaining in the pouch is then given by *m*(*t*) = *ρLwh*, where *ρ* is the density of the liquid, *h* is the average free surface height of the flow, and *L* is the length of the floor and by extension the flow^80^, (ii) the primary flow runs across only one wall of the tilted pouch (i.e. the floor). While the flow does partially interact with the side walls of the pouch, this can be neglected without significant error in our discussion.

The initial drainage rate is the same for the dry and oil-impregnated pouches at early time scales (0 s < *t* < 7 s). This is in agreement with previous works that observed that initial drainage was unaffected by the container shape or fluid properties^81–83^. This shows gravity is dominant over any surface effects in this brief period. By approximating the non-Newtonian ketchup as a Carreau fluid, its viscosity as a function of shear stress is given by^84,85^:

3 $$\mu ={\mu }_{{\rm{\infty }}}+({\mu }_{0}-{\mu }_{{\rm{\infty }}}){[1+{(\lambda \frac{du}{dy})}^{2}]}^{\frac{(n-1)}{2}}$$

where, *μ*_0_ is the zero-shear viscosity, *μ*_∞_ is infinite-shear viscosity, *λ* is the relaxation time, *du*/*dy* is the shear rate, and *n* is the power index whose value is *n* < 1 for shear-thinning fluids like ketchup. From Eq. 3, a shear-thinning non-Newtonian fluid behaves as a Newtonian fluid for small or large shear rates, while behaving as a shear-thinning fluid for intermediate shear rates. The high flow rate, and consequently high shear rate, of the initial regime of pouring therefore causes the ketchup to behave as a Newtonian fluid of constant viscosity *μ* → *μ*_∞_. The volumetric flow rate in this regime can be modeled as a simple gravity-driven Poiseuille flow of uniform thickness *h* = *h*(*t*) down an inclined plane (Fig. 8b):

4 $$Q\sim \frac{\rho g\,\sin (\alpha ){h}^{3}w}{{\mu }_{\infty }},$$

where *α* is the tilt angle of the pouch. Using $\dot{m}=\rho Q\sim {m}_{d}/t$, where *m*_*d*_ is the mass drained after some time *t*, it follows that:

5 $$\frac{{m}_{d}}{t}\sim \frac{{\rho }^{2}g\,\sin (\alpha ){h}^{3}w}{{\mu }_{\infty }},$$

Ali *et al*. has shown that at the end of the initial regime of drainage, the ratio of the mass remaining within the container to the initial mass remains fairly constant irrespective of container shape or fluid properties^83^. This mandates a constant value of *m*_*d*_/*t* for a time *t* corresponding to the end of this initial drainage regime, as the initial mass in the pouch was the same for both dry and infused pouches. Thus, from Equation (5), the scaling law $h\sim {t}^{-\mathrm{1/3}}$ is obtained. For a container of constant width, this also means that the ketchup mass remaining in the pouch scales as $m\sim {t}^{-\mathrm{1/3}}$, in excellent agreement with our experimental results for both dry and infused pouches.

In the second regime, corresponding to 10 s< *t* < 120 s for the dry pouch and 10 s < *t* < 40 s for the infused pouch, surface effects become important and the drainage rate from infused pouches ($m\sim {t}^{-0.4}$) greatly exceeds that of the dry ones ($m\sim {t}^{-0.2}$). At the moderate flow rates of this regime, the ketchup behaves like a shear-thinning fluid. The shear-thinning viscosity can be simply approximated as *μ* = *μ*_0_(∂*u*/∂*y*)^*n*−1 ^^84^. Flow in this regime can still be modeled as Poiseuille flow, but now *h* = *h*(*x*, *t*) is no longer uniform along the incline (Fig. 8b). Balancing shear stress and gravity:

6 $$\frac{\partial \tau }{\partial y}=\frac{dP}{dx}=-\,\rho g\,\sin \,\alpha ,$$

where *τ* = *μ*_0_(∂*u*/∂*y*)^*n*^ is the shear stress acting on the fluid. We can integrate Equation (6) using the zero shear stress boundary condition at the free surface (*τ*|_*y*=*h*_ = 0)^86^:

7 $$\tau ={\mu }_{0}{(\partial u/\partial y)}^{n}=\rho g\,\sin \,\alpha (h-y\mathrm{).}$$

Integrating again using the no-slip boundary condition: *u*(*x*, 0, *t*) = 0, Equation (7) can be integrated to obtain:

8 $$u(y)={[\frac{(\rho g\sin \alpha )}{{\mu }_{0}}]}^{\frac{1}{n}}\frac{n}{n+1}[{h}^{\frac{n+1}{n}}-{(h-y)}^{\frac{n+1}{n}}]\mathrm{.}$$

The average flow rate per unit width down the incline can be found by integrating $Q={\int }_{0}^{h}u(y)dy$, which yields:

9 $$Q\sim C{h}^{2n+\mathrm{1/}n},$$

where the *ρ*, *α* and *n* terms are collected as a constant *C*. By conservation of mass, ∂*Q*/∂*x* = −∂*h*/∂*t*. The power index for ketchup is known to be approximately *n* = 0.25^87^, such that the free surface equation can be expressed as:

10 $$\frac{\partial (C{h}^{6})}{\partial x}=\frac{\partial h}{\partial t},$$

From the chain rule, Eq. 10 becomes:

11 $$D{h}^{5}\frac{\partial h}{\partial x}=\frac{\partial h}{\partial t},$$

where *D* is a re-defined constant. Finally, this yields the scaling law $h\sim m\sim {(x/t)}^{\mathrm{1/5}}$, which is a perfect match with the measured rate of $m\sim {t}^{-0.2}$ for the dry pouch during this second regime.

Due to the presence of a thin oil-layer, in the oil-impregnated pouch, ketchup can slip at the infused wall^84,88^, resulting in a slip velocity of^21,84,89^:

12 $${u}_{s}=\frac{\mu }{\beta }{(\frac{\partial u}{\partial y})}_{y=0},$$

where *β* is the slip coefficient. Integrating Equation (6) with the new “slip” boundary condition, the velocity profile becomes^84,85,90^:

13 $$u(y)={[\frac{(\rho g\sin \alpha )}{{\mu }_{0}}]}^{\frac{1}{n}}\frac{n}{n+1}[{h}^{\frac{n+1}{n}}-{(h-y)}^{\frac{n+1}{n}}+{u}_{s}]\mathrm{.}$$

Note the extra term on the right-hand side compared to the drainage velocity over dry surfaces (Eq. 8), which is the likely cause of the enhanced drainage rate of the oil-impregnated pouch in this second regime ($m\sim {t}^{-0.4}$).

In the third regime, the ketchup draining from the pouch has diminished to become a thin film (Fig. 8b). As with the second regime, there is a marked improvement in the measured drainage rate for the oil-impregnated pouches ($m\sim {t}^{-0.8}$) compared to dry pouches ($m\sim {t}^{-0.5}$). Given the low flow rates of this third regime, the ketchup can be modeled as a Newtonian fluid with constant zero-shear viscosity *μ*_0_. For dry pouches, the flowing ketchup is akin to the classical thin-film flow of a Newtonian fluid down an inclined surface as shown by Jeffreys nearly a century ago^91^. A simple scaling analysis of the problem has been done by Quéré *et al*. which considers a balance between viscous resistance to the flow *μ*_0_*U*/*h*^2^ (where $U\sim {\rm{\Delta }}x/t$ is the average flow velocity) and gravitational force *ρg* to find the evolution of film thickness *h* with time^92^:

14 $$h\sim {({\mu }_{0}{\rm{\Delta }}x/\rho gt)}^{\mathrm{1/2}}\mathrm{.}$$

This results in a power law of $m\sim {t}^{-\mathrm{1/2}}$ for drainage in the third regime for the dry pouches, which again agrees with the experimental results. For the oil-impregnated pouches, the aforementioned slippage at the interface, in addition to the reduced hysteresis of any emerging contact lines, explains the larger drainage rate of $m\sim {t}^{-0.8}$ for the third regime of drainage. While it is possible that shear exerted by a test liquid flowing over an oil-impregnated surface can drain the oil^38^, the enhanced drainage of the ketchup from the infused pouches at this last stage of drainage indicates this is not happening appreciably for our system. This is intuitive given our very small effective pore size and the prior observation that shear-induced drainage is more problematic for larger, micro-scale pores^58^.

## Discussion

We have demonstrated that hydrocarbon-based polymers, such as polyethylene, can be easily impregnated with oils to create slippery packaging materials. While polymers such as polyethylene have long been considered impermeable to oils, we demonstrated that impregnation is possible when: (1) The polymer exhibits low density and degree of crystallinity (ex: ULDPE), (2) The lubricant is a vegetable oil for maximal chemical compatibility, and (3) The polymer layer is sufficiently thin (i.e. micrometric) to wick the oil inside of the molecular matrix by Washburn’s Law within a practical time scale (*t* < 1 s). The process of impregnation is as simple as coating a thin (10 *μ*m) pre-made polymer film with at least 0.5 g/m^2^ of oil and does not require any surface functionalization. Once the polymer film is impregnated with oil, it is highly slippery to a variety of test liquids such as water, ketchup, or yogurt. By using commercial-grade multilayer films with impermeable tie layers, the oil can be confined to one face of the film but not the reverse face. The oil-impregnation was quite stable, both to mechanical pressing against dry films and to long-term submersion under ketchup. When assembling the films into pouches, both the drainage rate and total drainage amount were significantly enhanced using the oil-impregnated films. Our recipe for easily imparting slippery and anti-fouling properties to commercial-grade extruded films should be highly useful for the packaging industry, particularly for food-release or pharmaceutical applications. More broadly, we expect that these findings will greatly expand the material palette used for creating slippery liquid-impregnated surfaces, as to date the polymer choices were restricted to expensive silicone-based polymers or complex multi-layer polymer assemblies on functionalized surfaces.

## Methods

### Infusion Method

Multilayer extruded commercial-grade polymer films (thickness ≈ 0.1 mm) obtained from Bemis Company, Inc. (Neenah, WI) were used as substrates for infusion. Most commonly, the top and bottom layers of the films were comprised of ULDPE (thickness ≈ 10 *μ*m), while the intermediate layers were a proprietary combination of highly impermeable polymers. For oil impregnation within the ULDPE polymer matrix, cottonseed oil (Sigma Aldrich) was used. Impregnation with oil was achieved using a motorized drawdown coater (ChemInstruments, EC-100) with the smallest size coating rod (size 0). First, 100–200 *μ*L of cottonseed oil (*ρ* = 925 kg/m^3^) was pipetted on the leading edge of the polymer film. Second, the motorized rod spread the oil uniformly across the top face of the film. After waiting for a few seconds to allow for the oil to impregnate the ULDPE, any excess oil atop the film was firmly wiped away with absorbent wipes (Kim wipes). For the control case where the oil did not impregnate the ULDPE, the same process was repeated but with 10 cSt silicone oil (Sigma Aldirch) instead of cottonseed oil. Other polymer films that were also used included medium density polyethylene (MDPE), cyclic olefin copolymer (COC), polypropylene (PP), or polyethylene terapthalate (PET) as the top layer.

### Details of Polymer Samples

The polymer samples used in this study were all manufactured from commercially available resins. The name, density, molecular weight, and degree of crystallinity of each polymer sample are shown in Table 1. The molecular weight of the ULDPE resin was not available, but its Melt Flow Index (MFI) is less than 0.5 g/10 min which is indicative of a very high molecular weight. Direct measurement of molecular weight distributions by gel permeation chromatography (GPC) or dilute solution viscometry are more involved and often do not correctly predict the molecular weight distribution especially for long chain branched polymers like our ULDPE sample which is an ethylene-butene copolymer. The Cyclic olefin copolymer (COC) used in this study is a cycloolefin copolymer of ethylene and norbornene (about 36 mol%). The degree of crystallinity for COC is an estimation based on the fact that this specific grade of resin is made of fully amorphous ethylene-norbornene backbone with a few percent LLDPE fraction which introduces the 2% crystallinity.

**Table 1:** Product name, density, molecular weight, and approximate degree of crystallinity for different polymer samples used in this study.

Polymer name	Resin name	Density (g/cc)	M_*w*_(g/mol)	Degree of crystallinity
ULDPE	Engage® HM 7387	0.87	Very High	16%
PP	SA861	0.90	280,000	46%
COC	Topas® 8007 F	1.01	104,000	∼2%
MDPE	NA272130X02	0.93	90,000	47%
PET	S2008	1.27	80,000	—

The blank values correspond to unknown information that is proprietary.

### Surface Characterization

The surface roughness for the dry ULDPE films was characterized by atomic force microscopy (AFM) (Multimode) analysis using a contact mode with a silicone nitride cantilever (Bruker). The scanning area was 20 *μ*m × 20 *μ*m. The roughness values did not change appreciably when the scan range was reduced to 5 *μ*m and 10 *μ*m. Two different methods were used to characterize the contact angle hysteresis of liquid droplets on dry or infused polymer samples. For water droplets, the shrink/swell method was employed using a contact angle goniometer (ramé-hart Model 590). A small (5 *μ*L) droplet was deposited on the test surface and its volume was increased/decreased in 0.2 *μ*L increments until the advancing/receding contact angle was obtained and measured by interfacing the camera with an automated software (DROPimage Advanced, ramé-hart). For highly viscous fluids like ketchup and yogurt, a 1.3 or 1.5 g dollop of the product was placed on the desired film, and the tilt angle was increased in 1° increments until finding the critical sliding angle where the dollop of ketchup/yogurt could slide down the surface. The sliding angle relates the gravitational and hysteresis forces, and is therefore correlated with contact angle hysteresis.

### Laser Scanning Confocal Microscopy

The oil impregnation and oil layer thickness were tested with the help of a laser scanning confocal microscope (Zeiss LSM 880). For better visualization, the cottonseed oil was dyed with a lipid soluble fluorescent dye (Bodipy FL C_5_, Thermo Fisher Scientific) at a concentration of 0.1 mg/mL. The oily side of the infused ULDPE film was fixed to a glass cover slip of 150 *μ*m thickness. A 40× objective was used which corresponds to a 1 *μ*m vertical resolution. The green noise around the bright green fluorescent oil layer is probably due to improper dye concentration within the oil. But even without the dye, the oil layer thickness found by the reflection mode came out to be similar in thickness which confirms that the dye concentration did not affect the oil thickness measurement.

### Wicking Test

To visualize oil wicking inside of ULDPE, a 100 *μ*L volume of oil (cottonseed oil or silicone oil) was mixed with 1.0% (w/v) petroleum fluorescent dye (Risk Reactor, DFSB-K175 UV Orange) using a vortex mixer. The fluorescent oil was placed in a (50 mm)^3^ open-top borosilicate cell (Spectrocell Inc.) to avoid optical distortion and illuminated with a UV light source (Risk Reactor, 52021(SLR-004-OL)). A vertically-oriented film of ULDPE was clamped over the fluorescent oil bath and the bath was raised on a z-stage until the bottom of the film was submerged. Images of the wicking front were taken in 5 min intervals for 9 hr by a Nikon D5300 camera. The displacement of the wicking front over time was measured using the Tracker software program. For both cottonseed oil and silicone oil, three wicking trials were performed.

### Pouch Fabrication

Two different types of pouch geometries were used in this study: three-walled pouches (two 18 cm × 12 cm side walls and a 6 cm × 12 cm base) and five-walled pouches (two 18 cm × 12 cm side walls, two 18 cm × 6 cm side walls and a 6 cm × 12 cm base). The pouches were constructed by bonding dry or infused multilayer ULDPE films together using a tabletop plastic film impulse sealer. The three-walled pouches were used for the long-term durability study (see Section 4.5) while the five-walled pouches were used to study the three regimes of pouch drainage (Section 2.4). The drainage performance of the three-walled and five-walled pouches were qualitatively similar; the five-walled pouches were made in an attempt to minimize confining edge effects while studying the dynamics of drainage.

### Durability Tests

The durability of oil-impregnated ULDPE films was assessed using two different types of tests.

*Pressing test*: Four multilayer films, all 6 cm × 6 cm in area, were stacked on top of each other. The top side of each film was comprised of an oil-impregnated ULDPE film layer, while the bottom side was a dry ULDPE layer. A 5 kg weight was placed on top of the stack for at least 24 hr, such that the impregnated ULDPE layers were in intimate contact with the opposing dry ULDPE layers. After removing the films from the stack, the contact angle hysteresis of water droplets on the impregnated ULDPE layers was measured using the shrink/swell method.

*Submersion test*: Three-walled pouches comprised of oil-impregnated ULDPE multilayer films were filled with ketchup and stored at room temperature for nearly two months (53 days). The drainage rate was then measured and compared to that of impregnated pouches that were drained immediately upon being filled with ketchup. There was no appreciable difference between the two, indicating the excellent stability of the cottonseed oil impregnated within the ULDPE layer even under prolonged submersion in the ketchup. See Supplementary Figure S3 in the Supplementary Information.

## Electronic supplementary material

Supplemental Information

Supplementary Movie M1

Supplementary Movie M2
