# Supplementary material for: Oil-Impregnated Hydrocarbon-Based Polymer Films
Source: Sci Rep. 2018 Aug 3;8:11698. doi: 10.1038/s41598-018-29823-7 (PMC6076315; doi:10.1038/s41598-018-29823-7)
Supplement: Supplementary file 1 — Supplemental Information [file 41598_2018_29823_MOESM1_ESM.pdf]

Supplemental Information: Oil-Impregnated Hydrocarbon-Based Polymer Films

Ranit Mukherjee[1], Mohammad Habibi[1], Ziad T. Rashed[1], Otacilio Berbert[2], Xiangke Shi[2], and Jonathan B. Boreyko[1]

[1]Macromolecules Innovation Institute, Department of Biomedical Engineering and Mechanics, Virginia Tech, Blacksburg, Virginia 24061, United States

[2]Bemis North America, Neenah, Wisconsin 54957

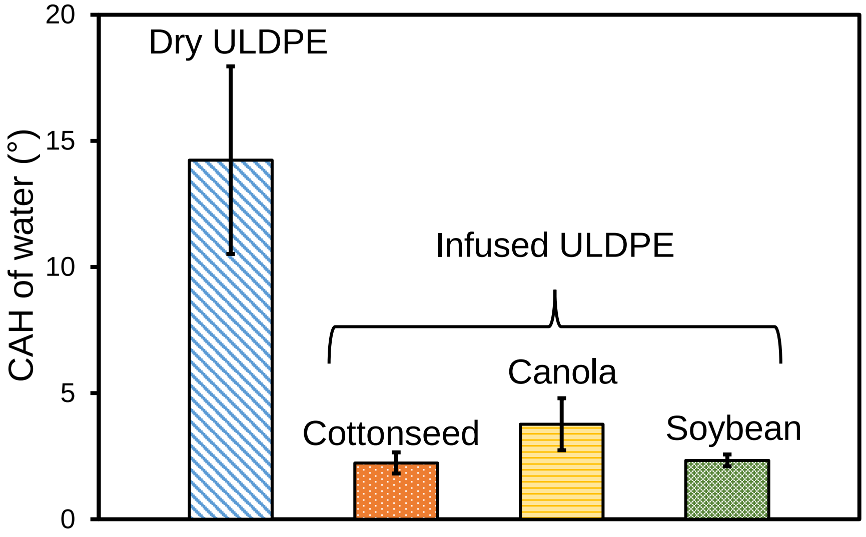

Supplementary Figure S1: When ULDPE films were impregnated with chemically compatible vegetable oils, the contact angle hysteresis (CAH) of water droplets was reduced to $<5°$.

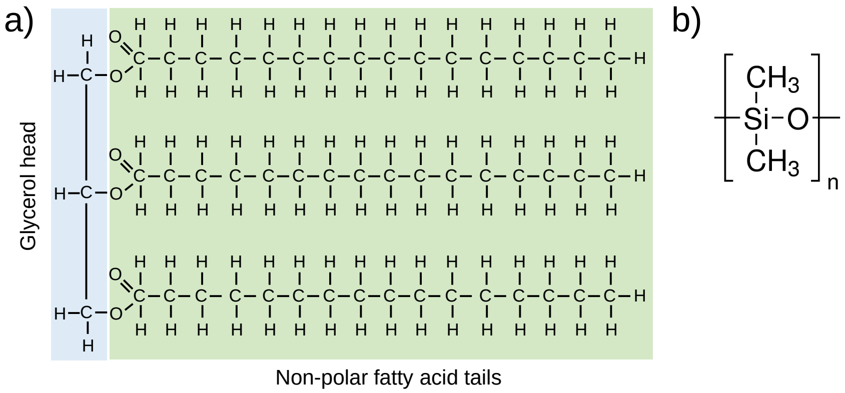

Supplementary Figure S2: a) Typical molecular structure of a saturated triglyceride. Triglycerides are the main components in vegetable fats and oils. A triglyceride molecule consists of three fatty acid molecules (shown in light green) connected to a glycerol backbone (shown in light blue). Due to the presence of these long hydrocarbon tails, triglycerides are non-polar in nature. b) A silicone oil (PDMS) molecular structure consists of Si-O bond and adjacent methyl groups. Due to the presence of this polar Si-O bond, silicone oil is polar in nature.

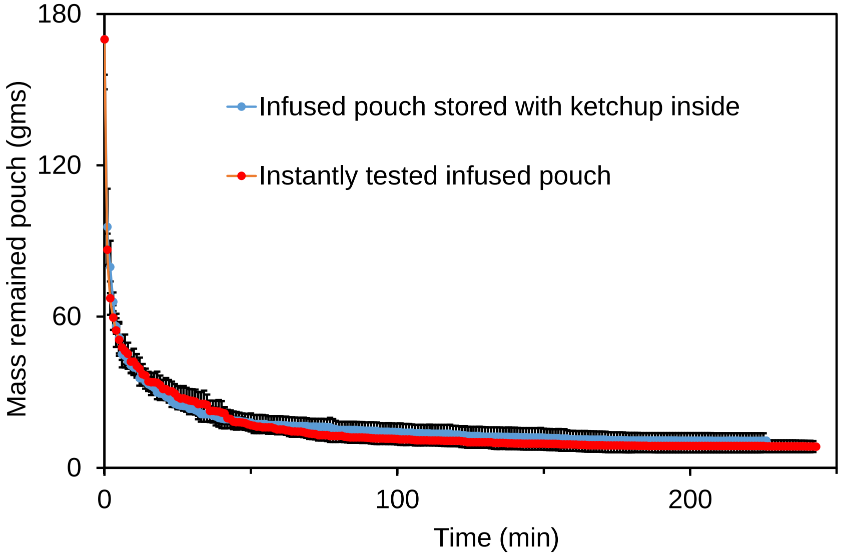

Supplementary Figure S 3: Drainage rates of ketchup being poured out of pouches comprised of oil-impregnated ULDPE films (see Section 4.5 in main text). There was no change in performance when draining pouches freshly filled with ketchup (red line) with pouches that were stored with ketchup for 53 days (blue line). This indicates the excellent stability of the impregnated oil within the ULDPE films. All error bars represent a standard deviation obtained from three trials.

1 Supplementary Videos

Supplementary Movie M1: Vertical wicking experiments on ULDPE films using baths of: a) cottonseed oil and b) silicone oil. The cottonseed oil readily wicks up the ULDPE film (black arrows in video), while no wicking occurs for the silicone oil. Both videos are constructed from images taken at 5 min intervals over 9 hr and played back at 5 fps (1,500 fast forward).

Supplementary Movie M2: Comparison of ketchup drainage from dry and oil-infused (right video) pouches with tilt angle of 45°. After the first 30 s of drainage shown here, there is visibly more ketchup in the dry pouch (left video) compared to the oil-impregnated pouch (right video).
